# Monte Carlo Tree Search-Based Recursive Algorithm for Feature Selection in High-Dimensional Datasets

**DOI:** 10.3390/e22101093

**Published:** 2020-09-29

**Authors:** Muhammad Umar Chaudhry, Muhammad Yasir, Muhammad Nabeel Asghar, Jee-Hyong Lee

**Affiliations:** 1 AiHawks, Multan 60000, Pakistan; 2 Department of Electrical and Computer Engineering, Sungkyunkwan University, Suwon 16419, Korea; 3 Department of Computer Science, University of Engineering and Technology Lahore, Faisalabad Campus, Faisalabad 38000, Pakistan; muhammadyasir@uet.edu.pk; 4 Department of Computer Science, Bahauddin Zakariya University, Multan 60000, Pakistan; nabeel.asghar@bzu.edu.pk

**Keywords:** feature selection, dimensionality reduction, R-MOTiFS, Monte Carlo Tree Search (MCTS), heuristic feature selection

## Abstract

The complexity and high dimensionality are the inherent concerns of big data. The role of feature selection has gained prime importance to cope with the issue by reducing dimensionality of datasets. The compromise between the maximum classification accuracy and the minimum dimensions is as yet an unsolved puzzle. Recently, Monte Carlo Tree Search (MCTS)-based techniques have been invented that have attained great success in feature selection by constructing a binary feature selection tree and efficiently focusing on the most valuable features in the features space. However, one challenging problem associated with such approaches is a tradeoff between the tree search and the number of simulations. In a limited number of simulations, the tree might not meet the sufficient depth, thus inducing biasness towards randomness in feature subset selection. In this paper, a new algorithm for feature selection is proposed where multiple feature selection trees are built iteratively in a recursive fashion. The state space of every successor feature selection tree is less than its predecessor, thus increasing the impact of tree search in selecting best features, keeping the MCTS simulations fixed. In this study, experiments are performed on 16 benchmark datasets for validation purposes. We also compare the performance with state-of-the-art methods in literature both in terms of classification accuracy and the feature selection ratio.

## 1. Introduction

With the abundance of huge data around, more sophisticated methods are required to handle it. Among the class of different techniques, feature selection is one that has gained much attention by the researchers, mainly because of the high dimensionality of big datasets. Such datasets usually comprise of high volumes of redundant or irrelevant dimensions/features. To eliminate such redundant or irrelevant features, feature selection techniques are deployed that select the optimal subset of features while maintaining the same or improved classification performance. Various fields where feature selection is playing a significant role includes, but is not limited to, machine learning [1,2], pattern recognition [3,4,5], statistics [6,7], and data mining [8,9]. However, maximizing the classification accuracy with the minimum possible feature set is not trivial. In fact, the tradeoff between the classification accuracy and the selected feature set size is an open challenge for the research community.

The literature divides the feature selection techniques as filter, wrapper, and embedded methods [10]. The filter-based methods use a proxy measure like correlation and information gain to rank the features in a feature subset [11,12,13]. They are usually fast and independent of any classification algorithm; however, their performance degrades in the existence of redundant features. In an attempt to tackle the issues associated with filter methods, the researchers have proposed information theoretic-based methods [14,15,16]. Wrapper methods use the stand-alone classification algorithm to measure the quality of the feature subsets [17,18]. Relatively, they are costly in terms of computational complexity but are still preferred over filter methods because of showing better classification performance. Embedded methods are different in a way that they perform feature selection as an integral part of the learning algorithm.

To search the feature space for an optimal feature subset within wrapper- or filter-based methods, various heuristics and meta-heuristic approaches have been developed, including the genetic algorithms (GA) [19,20], particle swarm optimization [21,22,23], and ant colony optimization [24]. Decision tree-based techniques have also been adopted by many researchers for feature selection. Wan et al. [25] applied the gradient-boosting decision trees to select the features from users’ comments about the items. Rao et al. [26] presented a framework integrating the artificial bee colony with gradient-boosting decision trees. Recently, the Monte Carlo Tree Search (MCTS)-based techniques have emerged and achieved a great success in the feature selection domain [27,28]. The MCTS is a lightweight search algorithm that combines the efficient tree search with random sampling [29]. The ability of MCTS to quickly place emphasis on the most valuable portions makes it suitable for huge search space problems [30]. It is pertinent to mention the feature selection algorithm, MOTiFS (Monte Carlo Tree Search-based Feature Selection), where the authors mapped the feature selection as a binary search tree and used MCTS for tree traversal to find the optimal set of features [27]. The MOTiFS showed remarkable performance as compared to the state-of-the art and other evolutionary feature selection methods. The inherent advantage of MOTiFS is the binary feature selection tree which shrinks the huge search space. However, the tradeoff between the performance/tree search and the number of simulations is challenging. The search tree might not meet the sufficient depth in a limited number of MCTS simulations, thus inducing bias towards randomness in feature subset selection. This intuition urged us and served as a catalyst for this study.

In this article, we extend the idea of MOTiFS and propose a recursive framework to take the full advantage of tree search for optimal feature selection. The idea is based on the intuition that the state space of every successor feature selection tree is smaller than that of its predecessor, thus increasing the impact of tree search in selecting best features, keeping the MCTS simulations fixed during each recursion. The algorithm starts with the full feature set F as an initial input and builds various feature selection trees in a series, each producing the best feature subset ($F_{best}$) as an output after S MCTS simulations. The output of each tree (the corresponding best feature subset) is injected as an input to the next tree in a series. This recursive procedure continues until the classification performance of best feature subset keeps on improving. The algorithm finally returns the optimal feature subset ($F_{optimal}$). Every successive recursion increases the impact of tree search because of the smaller feature space.

The proposed method is referred as R-MOTiFS (Recursive-Monte Carlo Tree search-based Feature Selection) and its performance is tested on 16 publicly available datasets. Considering its significant for high-dimensional datasets, we presented both the classification accuracy and the FSR (feature selection ratio) as the performance measures. The results are also compared with MCTS-based methods and other state-of-the-art methods which demonstrate the superiority of the proposed method.

The rest of the paper is structured as follows. The related work and the necessary background are presented in Section 2 (Background). The proposed method is explained in Section 3 (R-MOTiFS). The experimental details and results are provided in Section 4 (Experiment and results). Finally, we conclude the article in Section 5 (Conclusions).

## 2. Background

### 2.1. Related Work

Recently, a few researchers have tried to solve the feature selection problem using MCTS as a heuristic search strategy. In the reinforcement learning-based method FUSE, the authors used MCTS for searching the optimal policy [31]. The search tree is expanded exhaustively using all the features, thus increasing the state space exponentially. The authors implemented various heuristics to overcome the effect of huge branching factor. In the FSTD algorithm, the authors implemented a temporal difference-based strategy to traverse the huge search space to find the best feature subset [32]. A method for local feature subset selection is proposed in Reference [33]. The algorithm used MCTS to learn sub-optimal feature trees, by simultaneously partitioning the search space into different localities. The MCTS-based method to improve the relief algorithm is proposed in Reference [34]. The authors used the exhaustive tree with relief (a feature selection algorithm) as an evaluator to select the best feature subset. The Support Vector Machine is applied to check the accuracy of the obtained feature subset. Recently, a new algorithm, MOTiFS, was proposed, where the authors mapped the feature selection as a binary search tree and used MCTS to find the optimal feature subset [27]. The MOTiFS showed remarkable performance as compared to the state-of-the art and other evolutionary feature selection methods. The inherent advantage of MOTiFS is the binary feature selection tree which shrinks the huge search space. However, the tradeoff between the performance/tree search and the number of simulations is challenging. The search tree might not meet the sufficient depth in a limited number of MCTS simulations, thus inducing bias towards randomness in feature subset selection. This intuition urged us to study and devise the new algorithm which can effectively use the power of tree search along with the randomness in MCTS.

### 2.2. Monte Carlo Tree Search (MCTS)

MCTS is characterized as a heuristic search algorithm which uses lightweight random simulations to reach the final goal [29]. In a given domain, it finds the optimal decisions by taking the random samples in the decision space and building a search tree accordingly. The search tree is iteratively built until a termination condition is met. The state of the domain is represented by the nodes in the search tree, and the actions are represented by the directed links from a node to its child nodes. Each MCTS iteration consists of four sequential steps: Selection, Expansion, Simulation, and Backpropagation.

1. Selection: Starting from the root node, the algorithm traverses the tree by applying a recursive child selection policy until the urgent node is reached that represents a non-terminal state and has unvisited children.
2. Expansion: A tree is expanded by adding a new child node based on the set of actions available.
3. Simulation: A simulation is performed from the new child node according to the default policy to produce an approximate outcome.
4. Backpropagation: The reward of simulation is backed-up using the selected nodes to update the statistics of the tree.

Selection and Expansion stages are implemented using tree policy, whereas Simulation is controlled by default policy.

### 2.3. Upper Confidence Bounds for Trees (UCT)

The UCT algorithm is used to select the nodes during Selection and Expansion stages. The values of the nodes are approximated using Equation (1). At each level of a tree, the nodes are selected which have a largest approximated value.

(1) $$UCT_{v}=\;\frac{W_{v}}{N_{v}}+\;C\;\times \;\sqrt{\frac{2\;\times \;ln\left(N_{p}\right)}{N_{v}}}$$

where $N_{v}$ and $N_{p}$ represent the number of times nodes v and its parent p are visited, respectively. $W_{v}\;$represents the number of wining simulations (considering the games perspective) at node v. C is the exploration constant to keep the balance between exploration and exploitation.

## 3. R-MOTiFS (Recursive-Monte Carlo Tree Search-Based Feature Selection)

R-MOTiFS is a recursive framework for feature selection where multiple feature selection trees are built iteratively in a recursive fashion. The state space of every successor feature selection tree is smaller than that of its predecessor, thus increasing the impact of tree search in selecting best features, keeping the MCTS simulations fixed. Given a full feature set F as an initial input, various trees are built in a series, each producing the best feature subset as an output after S MCTS simulations. The output of each tree is injected as an input to the next tree in a series. This recursive procedure continues until the base condition is satisfied, and finally returns the optimal feature subset. The detailed algorithm is explained in the following sub-sections. Table 1 summarizes the notations used throughout the text.

### 3.1. The Recursive Procedure

The algorithm starts with a full feature set (F) and calls a recursive procedure to find the best feature subset ($F_{best}$) for n possible recursions. During each recursion, a feature selection tree is constructed following the S MCTS-based simulations to find the best feature subset.

**Figure 1:**
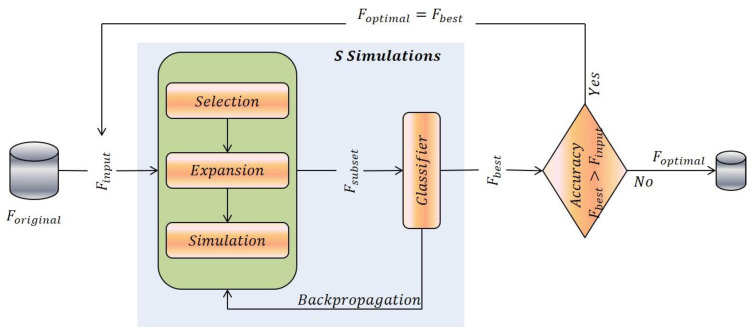
The proposed method, Recursive-Monte Carlo Tree Search-Based Feature Selection (R-MOTiFS).

Assuming the number of recursions from 0 to n, represented as 0, 1, 2, …, i, j, …, n, for the $i_{th}$ recursion, a feature set $F^{i}$ is provided as an input (at the root node) and the search tree is incrementally built following the tree and default policies. After S MCTS simulations, the best feature subset $F_{best}^{i}$ is found (such that $F_{best}^{i}$is the subset of $F^{i}$). Conditioned on improved classification performance of $F_{best}^{i}$ as compared to $F^{i}$, the best feature subset $F_{best}^{i}$ is designated as the optimal feature subset ($F_{optimal}$) and fed into the $j_{th}$ (next) recursion as an input (i.e., $F^{j}\;$= $F_{best}^{i}$) to generate a successor feature selection tree, producing the best feature subset $F_{best}^{j}$. This recursive procedure continues until the base condition, $Acc\left(F_{best}^{j}\right)<Acc\left(F_{best}^{i}\right)$, is satisfied (i.e., the best feature subset found in the $j_{th}$ recursion degrades the classification accuracy as compared to the best feature subset found in the $i_{th}$ recursion (also designated as $F_{optimal}$). The algorithm finally returns the optimal feature subset, $F_{optimal}$. The procedure is graphically represented in Figure 1.

The rest of this section is dedicated to the detailed description of the search procedure including the feature selection tree, feature subsets generation, and the evaluation function during each recursion.

### 3.2. Feature Selection Tree

A feature can have two states: either it is selected or not in the feature subset. Based on this principle, a feature selection tree is constructed, which is defined as [27]:

**Definition** **1:** *For a feature set, $F=\left\{f_{1},\;f_{2},\;\ldots ,\;f_{i},\;\ldots ,\;f_{n}\right\}$, the feature selection tree is a tree satisfying the following conditions:*

*1.*  *The root is $\emptyset_{0}$, which means no feature is selected yet.*
*2.*  *Any node at level*i−1*has two children,*$f_{i}$*and*$\emptyset_{i}$*, where*0<i<n.

**Figure 2:**
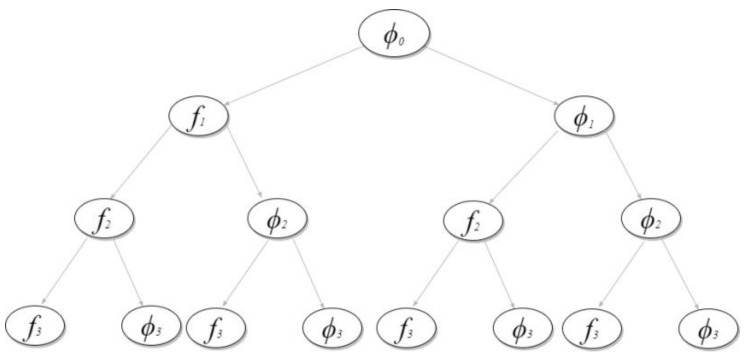
Feature selection tree where $F=\left\{f_{1},\;f_{2},\;f_{3}\right\}.$

*where nodes* $f_{i}$ *and* $\emptyset_{i}$ *represent the feature states: inclusion or exclusion of the corresponding feature $f_{i}$ in the feature subset, respectively. Any path from the root node to one of the leaves represents a feature subset. So, the goal is to find a path which gives the best reward (accuracy). We used MCTS for tree construction and traversal, and finally chose the path with best accuracy. The features in a chosen path form a feature subset, referred as best feature subset, F_best_, for the current feature selection tree. Figure 2 shows the complete tree where $F=\left\{f_{1},\;f_{2},\;f_{3}\right\}$.*

### 3.3. Feature Subset Generation

Starting with a root node, a search tree is incrementally constructed by adding nodes representing the feature states. During each simulation, a feature subset is generated following tree (Selection and Expansion stages) and *default* (Simulation stage) policies.

At Selection and Expansion stages, features are selected based on the tree policy, where the modified form of the UCT algorithm, as shown in Equation (2), is used to decide on the inclusion or exclusion of the features in a feature subset. Out of the two children $f_{i}$ and $\emptyset_{i}$ at level i, if Equation (2) gives a high score to $f_{i}$ then feature $f_{i}$ is included in the feature subset, otherwise it is not included.

(2) $$UCT_{v_{j}}=\;\max\left(Q_{v_{j}}\right)+\;C\;\times \sqrt{\;\frac{2\;\times \;ln\left(N_{v_{i}}\right)}{N_{v_{j}}}}$$

where $\max\left(Q_{v_{j}}\right)\;$ is the maximum reward at the node $v_{j}$ and C>0 is a constant. $N_{v_{j}}$ and $N_{v_{i}}$ represent number of times nodes $v_{j}$ and its parent $\;v_{i}$ are visited, respectively.

The tree policy controls the tree traversal (selection of feature states) until the most urgent node (a node which is non-terminal and has an unexpanded child) is expanded. From this point to the leaf node, a random simulation is run where the features are included in the feature subset following the default policy. This unique combination of tree search and random sampling speeds up the process of finding the best feature subset without expanding and traversing the whole feature selection tree.

### 3.4. Reward Calculation and Backpropagation

As an evaluation metric to measure the goodness of the feature subset, we used the classification accuracy, which is also referred to as a simulation reward $Q_{simulation}$ for the current chosen path. The simulation reward is propagated backwards through the current path to update the search tree.

(3) $$Q_{simulation}=ACC_{classifier}\left(F_{subset}\right)$$

where $ACC_{classifier}\left(F_{subset}\right)$ represents the accuracy of the classifier on the current feature subset, $F_{subset}$. If the accuracy of the current feature subset is better than the previous best, then the current feature subset becomes the best feature subset. This process continues until stopping criteria is met, i.e., the fixed number of simulations, *S*.

In this study, we used the K-NN (K-Nearest Neighbors) classifier to evaluate the feature subset. K-NN is generalized as an efficient and simple learning method which has proven its significance in the literature [35,36,37]. The detailed algorithm of our proposed method is presented below as Algorithm 1.
**Algorithm 1** The R-MOTiFS AlgorithmLoad dataset and preprocessInitialize SCALAR, BUDGET *//Scaling factor & Number of MCTS simulations (hyper parameters)***function** R-MOTiFS (*featuresList*)    create *rootNode*    *maxReward, bestFeatureSubset* ← UCTSEARCH (*rootNode*)    **if**
*maxReward* is greater than *optimalReward*
**then**      *optimalReward ← maxReward*      *optimalFeatureSubset ← bestFeatureSubset*      R-MOTiFS (*bestFeatureSubset*)**else**      **return** (*optimalReward, optimalFeatureSubset*)**function** UCTSEARCH (*rootNode*)    Initialize *maxReward, bestFeatureSubset*    **while** within computational budget **do**      *frontNode* ← TREEPOLICY (*rootNode*)      *reward, featureSubset* ← DEFAULTPOLICY (*frontNode.state*)      BACKUP (*frontNode, reward*)       **if**
*reward* is greater than *maxReward*
**then**        *maxReward ← reward*        *bestFeatureSubset ← featureSubset*    **return** (*maxReward, bestFeatureSubset*)**function** TREEPOLICY (*node*)    **while**
*node* is non-terminal **do**      **if**
*node* not fully expanded **then**        **return** EXPAND (*node*)      else        *node* ← BESTCHILD (*node, SCALAR*)    **return**
*node***function** EXPAND (*node*)    choose *a*
∈ untried actions from *A*(*node.state*)    add a *newChild* with *f*(*node.state, a*)    **return**
*newChild***function** BESTCHILD (v*,*
 C)    $\mathrm{return}\;\underset{v^{\prime }\in \;\mathrm{children}\;\mathrm{of}\;v}{\max}\max\left(Q_{v^{\prime }}\right)+C\sqrt{\frac{2\;\times \;ln\left(v.visits\right)}{v^{\prime }.visits}}$**function** DEFAULTPOLICY (*state*)    **while**
*state* is non-terminal **do**      choose *a*∈
*A*(*state*) uniformly at random      *state ← f*(*state, a*)**traverse***state.path*      **if**
*a_i_* is equal to *f_i+1_*
**then**        *featureSubset ←* INCLUDE (f_i+1_)    *reward* ← REWARD (*featureSubset*)    **return** (*reward, featureSubset*)**function** BACKUP (*node, reward*)    **while**
*node* is not null **do**      *node.visits ← node.visits* + 1      **if**
*reward* > *node.reward*
**then**        *node.reward ← reward*      *node ← node.parent*    **return**

## 4. Experiment and Results

### 4.1. Datasets

We experimented on 16 publicly available datasets downloaded from UCI [38] and LIBSVM [39]. The datasets are taken from different application domains including medical science, molecular biology, object recognition, email filtering, handwritten digits classification, etc. The details of datasets are summarized in Table 2. The datasets taken are of varying dimensions and sizes with a minimum of 20 feature dimensions.

### 4.2. Experimental Setting

The two parameters involved in our proposed method are the “Scaling factor” and the “Termination criteria”. The “Scaling factor”, C, in Equation (2) is set to 0.1. The “Termination criteria” refers to the number of simulations S performed during each recursion. We set the value of S to 1000. For the classifier, K-NN, we set the value of K to 5.

We used 10-fold cross-validation, where 9-folds were used as the training and validation set and the remaining 1-fold as a test set. Hence, each fold is used exactly once as a test set. Being the heuristic method, we performed 5 independent runs on every dataset and reported the average results.

### 4.3. Results and Comparisons

This section presents the comparison of R-MOTiFS with MOTiFS (Monte Carlo Tree Search based Feature Selection), H-MOTiFS (Hybrid-Monte Carlo Tree Search based Feature Selection), and other state-of-the-art methods.

#### 4.3.1. Comparison with MOTiFS and H-MOTiFS

Table 3 and Table 4 provide the comparison of our proposed method, R-MOTiFS, with MOTiFS and H-MOTiFS. Table 3 provides the detailed comparison w.r.t the classification accuracy and the number of selected features, whereas the overall comparison is provided in Table 4 in terms of a unique measure, called the feature selection ratio.

Comparing R-MOTiFS with MOTiFS in terms of classification accuracy in Table 3, it is clear that R-MOTiFS shows the best performance on 11 out of 16 datasets, namely “Spambase”, “Ionosphere”, “Arrhythmia”, “Multiple features”, “Waveform”, “DNA”, “Hill valley”, “Musk 1”, “Coil20”, “Kr-vs-kp”, and “Spect”. On one dataset, “Orl”, the accuracy of R-MOTiFS is equal to the MOTiFS. Comparing with H-MOTiFS, it can be seen that R-MOTiFS has the best performance on four datasets, namely “Spambase”, “Arrhythmia”, “Multiple Features”, and “Musk 1” w.r.t classification accuracy. However, on other datasets, R-MOTiFS shows nearly equal or less classification accuracy as compared with H-MOTiFS.

The performance of R-MOTiFS is remarkable in terms of the selected features. The number of selected features is reduced by a huge margin, as compared to MOTiFS and H-MOTiFS algorithms, on almost all the datasets. Particularly, on high-dimensional datasets like “Arrhythmia”, “Multiple features”, “DNA”, “Hill valley”, “Musk 1”, “Coil20”, and “Orl”, the extensive reduction in features with the improved or nearly equal classification performance shows the significance of R-MOTiFS. This evidence endorses the intuition that in successive feature selection trees, the impact of tree search increases with a reduction in search space, thus increasing the performance overall.

Considering the abundance of high-dimensional datasets, we understand that only accuracy is not the sufficient measure to estimate the performance of a classifier. The selected feature set size is as significant as the classification accuracy. The ultimate objective is to maximize the accuracy with the minimum possible feature set size. In fact, it is hard to assess the overall performance by treating the two (classification accuracy and the selected feature set size) individually. One unique metric to check the combined effect of the classification accuracy and the selected feature set size is referred to as FSR (feature selection ratio):

(4) FSR=Accuracy/No. of Selected Features

The comparison of R-MOTiFS with MOTiFS and H-MOTiFS, in terms of FSR, is provided in Table 4. It can be clearly observed that R-MOTiFS outperforms MOTiFS on all the datasets with a huge margin. While comparing with H-MOTiFS, our proposed method R-MOTiFS shows best performance on 10 datasets, including all high-dimensional datasets, namely “Spambase”, “Ionosphere”, “Arrhythmia”, “Multiple features”, “DNA”, “Hill valley”, “Musk 1”, “Coil20”, “ORL”, and “Lung-discrete”. It clearly demonstrates the superiority of our proposed method, R-MOTiFS.

The standard deviation of five independent runs of R-MOTiFS on each dataset is also reported in Table 3. The negligible values indicate the stability of our proposed method.

#### 4.3.2. Comparison with State-Of-The-Art Methods

Table 5 provides the comparison of our proposed method, R-MOTiFS, with other evolutionary and state-of-the-art methods. The comparison methods were chosen to maintain the diversity and quality of the works reported. Examining Table 5 reveals the significance of the proposed method.

Let us discuss the pairwise comparison first. Comparing with GA, our proposed method, R-MOTiFS, shows better classification accuracy on 13 out of 16 datasets, namely “Spambase”, “Ionosphere”, “Arrhythmia”, “Multiple ft.”, “Waveform”, “WDBC”, “GermanNumber”, “DNA”, “Musk 1”, “Coil20”, “ORL”, “Lung_discrete”, and “Spect”. Comparing with SFSW on 11 datasets, R-MOTiFS performs best on 9 datasets. In the comparison between R-MOTiFS and E-FSGA, performed on 8 datasets, R-MOTiFS outperformed on 6 datasets. Comparing with PSO (4-2) on 7 datasets, R-MOTiFS outperformed on all the datasets, except one dataset, “Hillvalley”. R-MOTiFS shows top performance on 6 datasets compared with WoA and WoA-T over 7 datasets.

Let us look at Table 5 collectively. Among the 16 datasets compared, R-MOTiFS outperformed all the other methods on 10 datasets, namely “Ionosphere”, “Arrhythmia”, “Multiple features”, “German number”, “DNA”, “Musk 1”, “Coil20”, “Orl”, “Lung_discrete”, and “Spect”. Along with achieving high accuracy, R-MOTiFS selected less features as compared to other methods, in most of the cases. On four datasets, namely “Spambase”, “Waveform”, “WDBC”, and “Kr-vs-kp”, R-MOTiFS ranked second in a row. There were only two datasets, “Sonar” and “Hill valley”, where R-MOTiFS stood third or less as compared to all the other methods.

We further provide the comparison of R-MOTiFS with other state-of-the-art methods in terms of FSR. Examining Table 6 pairwise, we can see that R-MOTiFS outperformed GA, SFSW, WoA, and WoA-T on all the corresponding, 16, 11, 7, and 7, datasets, respectively. There is only one comparative method, PSO (4-2), where R-MOTiFS could not beat on all the datasets. This is mainly because PSO (4-2) tends to select a very small number of features with compromised accuracy, resulting in high FSR.

Examining Table 6 collectively reveals that on 12 out of 16 datasets, R-MOTiFS ranked first as compared to all other methods. On the remaining 4 datasets, R-MOTiFS maintained the second position overall. It clearly demonstrates the overall superiority of our proposed method.

Summing up the performance of R-MOTiFS, it is evident that R-MOTiFS showed outstanding results both in terms of high classification accuracy and reduced feature dimensions. Comparison with MCTS-based methods (MOTiFS and H-MOTiFS) and other state-of-the-art methods showed the significance of the proposed method. In a limited number of simulations scenario, the randomness in MCTS simulations could be the reason for noise in the basic MOTiFS algorithm, thus inclining it towards selecting a high number of features, relatively, particularly on high-dimensional datasets. However, in R-MOTiFS, the successive feature selection trees reduced the impact of randomness by focusing on the tree search in a recursive fashion, thus improving the performance by a great margin. The experimental results demonstrate the effectiveness of R-MOTiFS and establish the strong recommendation of its use for feature selection in various application domains.

#### 4.3.3. Non-Parametric Statistical Tests

In order to check the statistical significance of our proposed method, we perform the Wilcoxon Signed-Rank and Friedman tests using the FSR values reported in Table 4 and Table 6 above.

For the pairwise comparison of R-MOTiFS with the other methods, Wilcoxon Signed-Rank test was performed with a p-value of 0.05 and the results are reported in Table 7. The high R+ results (as compared to R–) in each row indicate the superiority of R-MOTiFS as compared to all the other methods, except the PSO (4-2). As mentioned above, this is mainly because PSO (4-2) tends to select a very small number of features with compromised accuracy, resulting in high FSR. This fact can be observed by looking at Table 5, where PSO (4-2) shows very low accuracy values in most of the cases, along with selecting a very small number of features. Observing the p or w values reveals that the null hypothesis is rejected against the comparison methods, MOTiFS, GA, SFSW, WoA, and WoA-T; thus, the results are statistically significant at a p-value of 0.05 against these methods.

To check the statistical significance overall, we performed the Friedman test using the FSR values reported. We compared the five methods (R-MOTiFS, H-MOTiFS, MOTiFS, SFSW, and GA) on 11 common datasets, namely “Spambase”, “Ionosphere”, “Arrhythmia”, “Multiple ft.”, “Waveform”, “WDBC”, “GermanNumber”, “DNA”, “Sonar”, “Hillvalley”, and “Musk 1”. We did not include PSO (4-2), WoA, and WoA-T in the comparison because of the lower number of common datasets. Examining Table 8 reveals that R-MOTiFS ranked first among all the comparison methods. Also, the p-value was 0.061, which shows that the results are significant at p<0.10.

## 5. Conclusions

In this paper, we proposed the MCTS-based recursive algorithm for feature selection to reduce the complexity and high dimensionality of data. The proposed algorithm constructed the multiple feature selection trees in a recursive fashion such that the state space of every successor tree was less than its predecessor, thus maximizing the impact of tree search in selecting the best features, keeping the number of MCTS simulations fixed. Experiments were carried out on 16 benchmark datasets and results were also compared with the state-of-the-art methods in the literature. Considering their significance for high-dimensional datasets, we presented both the classification accuracy and the FSR (feature selection ratio) as the performance measures. Besides achieving high classification accuracy, our proposed method significantly reduced the dimensionality of datasets, thus making it a perfect candidate to be used in different application domains.

## Figures and Tables

**Table 1:** Notations used to explain the proposed method.

Notation	Interpretation
F	Original feature set
$F^{i}$	Input feature set in $i_{th}$ recursion
$F_{best}^{i}$	Best feature subset in $i_{th}$ recursion
$v_{i}$	Node v at tree level i
$N_{v_{i}}$	Number of times node $v_{i}$ is visited
$Q_{simulation}$	Simulation reward

**Table 2:** Summary of the selected datasets.

#	Dataset	No. of Features	No. of Instances	No. of Classes
1	Spambase	57	4701	2
2	Ionosphere	34	351	2
3	Arrhythmia	195	452	16
4	Multiple Features	649	2000	10
5	Waveform	40	5000	3
6	WBDC	30	569	2
7	German number	24	1000	2
8	DNA	180	2000	2
9	Sonar	60	208	2
10	Hillvalley	100	606	2
11	Musk 1	166	476	2
12	Coil20	1024	1440	20
13	Orl	1024	400	40
14	Lung_Discrete	325	73	7
15	Kr-vs-kp	36	3196	2
16	Spect	22	267	2

**Table 3:** Comparison of R-MOTiFS with MOTiFS and H-MOTiFS. Best results in each row are in bold.

Dataset	Accuracy Number of Selected Features		
	R-MOTiFS	MOTiFS [25]	H-MOTiFS [26]
Spambase	**0.915** ± 0.003 15.5	0.907 31.5	0.907 18.0
Ionosphere	0.890 ± 0.008 4.72	0.889 12.3	**0.892** 7.0
Arrhythmia	**0.678** ± 0.008 12.3	0.650 94.4	0.640 40.0
Multiple features	**0.982** ± 0.002 110.5	0.980 321.8	**0.983** 195.0
Waveform	0.817 ± 0.005 14.4	0.816 19.4	**0.823** 12.0
WDBC	0.962 ± 0.002 12.6	**0.967** 15.4	0.964 6.0
German Number	0.718 ± 0.014 8.6	0.725 11.5	**0.728** 8.0
DNA	0.893 ± 0.002 12.2	0.810 89.3	**0.905** 18.0
Sonar	0.834 ± 0.003 14.1	**0.850** 28.9	0.836 12.0
Hill valley	0.552 ± 0.016 9.5	0.535 45.2	**0.566** 10.0
Musk 1	**0.853** ± 0.010 32.7	0.852 81.3	0.850 50.0
Coil20	0.981 ± 0.009 81.6	0.980 505.4	**0.989** 308.0
Orl	0.862 ± 0.011 135.3	0.862 498.3	**0.883** 308.0
Lung_discrete	0.807 ± 0.006 41.0	0.810 154.8	**0.823** 98.0
Kr-vs-kp	0.964 ± 0.005 16.2	0.961 20.1	**0.975** 8.0
Spect	0.813 ± 0.008 8.7	0.809 10.3	**0.817** 7.0

**Table 4:** Comparison of R-MOTiFS with MOTiFS and H-MOTiFS w.r.t FSR (feature selection ratio). Best results in each row are in bold.

DataSet	FSR		
	R-MOTiFS	MOTiFS [25]	H-MOTiFS [26]
Spambase	**0.059**	0.029	0.050
Ionosphere	**0.188**	0.072	0.127
Arhythmia	**0.055**	0.007	0.016
Multiple ft.	**0.009**	0.003	0.005
Waveform	0.057	0.042	**0.068**
WDBC	0.076	0.063	**0.161**
GermanNumber	0.083	0.063	**0.091**
DNA	**0.073**	0.009	0.050
Sonar	0.059	0.029	**0.069**
HillValley	**0.058**	0.012	0.056
Musk 1	**0.026**	0.010	0.017
Coil20	**0.012**	0.002	0.003
ORL	**0.006**	0.002	0.003
Lung_discrete	**0.020**	0.005	0.008
Kr-vs-Kp	0.060	0.048	**0.122**
Spect	0.093	0.079	**0.116**

**Table 5:** Comparison of R-MOTiFS with other methods. Best results in each row are bold and underlined. The second-best results in each row are in bold. “-” is placed wherever information is not available.

Dataset	Accuracy, Number of Selected Features						
	R-MOTiFS	GA	SFSW [40]	E-FSGA [41]	PSO (4-2) [42]	WOA [43]	WOA-T [43]
Spambase	**0.915** 15.5	0.910 26.0	0.885 26.0	**0.922**	-	-	-
Ionosphere	**0.891** 4.72	0.875 11.0	0.883 11.5	0.862	0.873 3.3	**0.890** 21.5	0.884 20.2
Arhythmia	**0.678** 12.2	0.635 101.0	**0.658** 100.0	-	-	-	-
Multiple Feat	**0.982** 110.5	0.976 339.0	**0.979** 270.0	0.945	-	-	-
Waveform	**0.818** 14.4	0.817 18.0	**0.837** 16.0	-	-	0.713 33.2	0.710 33.7
WDBC	**0.962** 12.62	0.961 18.0	0.941 13.5	**0.969**	0.940 3.5	0.955 20.8	0.950 20.6
GermanNumber	**0.718** 8.62	**0.715** 9.0	0.713 10.5	-	0.685 12.8	-	-
DNA	**0.893** 12.16	**0.860** 87.0	0.831 71.8	-	-	-	-
Sonar	0.834 14.1	**0.856** 26.0	0.827 20.0	0.808	0.782 11.2	0.854 43.4	**0.861** 38.2
HillValley	0.552 9.52	0.564 32.0	**0.575** 40.0	-	**0.578** 12.2	-	-
Musk 1	**0.852** 32.7	0.840 75.0	0.815 59.3	-	**0.849** 76.5	-	-
Coil20	**0.983** 81.65	**0.982** 462.0	-	0.892	-	-	-
ORL	**0.860** 135.32	**0.858** 571.0	-	0.622	-	-	-
Lung discrete	**0.807** 41.0	**0.800** 115.0	-	0.713	0.784 6.7	0.730	0.737
Kr-vs-Kp	**0.964** 16.2	**0.970** 17.0	-	-	-	0.915 27.9	0.896 26.7
Spect	**0.813** 8.72	**0.805** 11.0	-	-	-	0.788 12.1	0.792 11.5

GA: Genetic Algorithm. SFSW: Simultaneous Feature Selection and Weighing. E-FSGA: Ensemble Feature Selection using bi-objective Genetic Algorithm. PSO(4-2):Particle Swarm Optimization. WoA: Whale Optimization Algorithm. WoA-T: Whale Optimization Algorithm-Tournament selection.

**Table 6:** Comparison of R-MOTiFS with other methods w.r.t FSR (feature selection ratio). Best results in each row are bold and underlined. The second-best results in each row are in bold.

Dataset	FSR					
	R-MOTiFS	GA	SFSW [40]	PSO (4-2) [42]	WOA [43]	WOA-T [43]
Spambase	**0.059**	**0.035**	0.034	-	-	-
Ionosphere	**0.188**	0.079	0.077	**0.264**	0.041	0.044
Arhythmia	**0.055**	**0.006**	**0.006**	-	-	-
Multiple Feat.	**0.009**	0.003	**0.004**	-	-	-
Waveform	**0.057**	0.045	**0.052**	-	0.021	0.021
WDBC	**0.076**	0.053	0.070	**0.268**	0.046	0.046
GermanNumber	**0.083**	**0.079**	0.068	0.053	-	-
DNA	**0.073**	**0.010**	0.011	-	-	-
Sonar	**0.059**	0.033	0.041	**0.069**	0.020	0.022
HillValley	**0.058**	0.017	0.014	**0.047**	-	-
Musk 1	**0.026**	0.011	**0.014**	0.011	-	-
Coil20	**0.012**	**0.002**	-	-	-	-
ORL	**0.006**	**0.002**	-	-	-	-
Lung_discrete	**0.020**	0.007	-	**0.117**	0.010	0.011
Kr-vs-Kp	**0.060**	**0.057**	-	-	0.033	0.034
Spect	**0.093**	**0.073**	-	-	0.065	0.069

**Table 7:** Results of the Wilcoxon Signed-Rank Test.

R-MOTiFS vs.	R+	R–	p-Value	w-Value
MOTiFS	136	0	0.0004	0
H-MOTiFS	72.5	63.5	0.8181	63.5
GA	136	0	0.0004	0
SFSW	66	0	0.0033	0
PSO (4-2)	9	19	NA	9
WoA	28	0	NA	0
WoA-T	28	0	NA	0

**Table 8:** Results of Friedman Test.

Methods	Rank
R-MOTiFS	1.36
H-MOTiFS	1.64
MOTiFS	4.64
SFSW	3.45
GA	3.72
	p=0.061
